# Fundamentals of deep brain stimulation for Parkinson's disease in clinical practice: part 2

**DOI:** 10.1055/s-0044-1786037

**Published:** 2024-04-23

**Authors:** Mariana Moscovich, Camila Henriques de Aquino, Murilo Martinez Marinho, Lorena Broseghini Barcelos, André C. Felício, Matthew Halverson, Clement Hamani, Henrique Ballalai Ferraz, Renato Puppi Munhoz

**Affiliations:** 1 Christian-Albrechts University, Department of Neurology, Kiel, Germany.; 2 University of Calgary, Cumming School of Medicine, Department of Clinical Neurosciences, Calgary, AB, Canada.; 3 University of Calgary, Hotchkiss Brain Institute, Calgary, AB, Canada.; 4 Universidade Federal de São Paulo, Escola Paulista de Medicina, Departamento de Neurologia e Neurocirurgia, São Paulo SP, Brazil.; 5 Hospital Israelita Albert Einstein, São Paulo SP, Brazil.; 6 University of Utah, Department of Neurology, Salt Lake City, Utah, United States.; 7 University of Toronto, Sunnybrook Hospital, Toronto, ON, Canada.; 8 University of Toronto, Toronto Western Hospital, Toronto, ON, Canada.

**Keywords:** Deep Brain Stimulation, Parkinson Disease, Neurosurgery, Estimulação Encefálica Profunda, Doença de Parkinson, Neurocirurgia

## Abstract

The field of neuromodulation has evolved significantly over the past decade. Developments include novel indications and innovations of hardware, software, and stimulation techniques leading to an expansion in scope and role of these techniques as powerful therapeutic interventions. In this review, which is the second part of an effort to document and integrate the basic fundamentals and recent successful developments in the field, we will focus on classic paradigms for electrode placement as well as new exploratory targets, mechanisms of neuromodulation using this technique and new developments, including focused ultrasound driven ablative procedures.

## Resumo

O campo da neuromodulação evoluiu significativamente na última década. Esse progresso inclui novas indicações e inovações de *hardware* , *software* e técnicas de estimulação, levando a uma expansão das áreas clínicas cobertas e no papel dessas técnicas como intervenções terapêuticas eficazes. Nesta revisão, que é a segunda parte de um esforço para documentar e integrar os fundamentos básicos e os desenvolvimentos recentes e bem-sucedidos no campo, vamos nos concentrar em paradigmas clássicos para colocação de eletrodos, bem como em novos alvos exploratórios, mecanismos de neuromodulação usados por esta técnica e novos desenvolvimentos, incluindo procedimentos ablativos orientados por ultrassom focalizado.

## INTRODUCTION

The introduction of fully implanted deep brain stimulation (DBS) systems in clinical practice in the 1970s led to a paradigm shift in the treatment landscape of a variety of neurological and psychiatric disorders, including pain, epilepsy, and movement disorders, such as tremors, Parkinson's disease (PD) and dystonia. 1 Over these decades, DBS became a well-established therapeutic option, and its' indications and scope of use broadened, leading an estimated number of DBS systems implants worldwide to reach more than 200.000 in 2021. 2 Along with its' increased recognition as a powerful treatment choice, the complexity and sophistication of newer systems continued to expand substantially.

This review is the second part of a broad effort to document and integrate the basic fundamentals and recent successful developments in the field. This part of the review will focus on classic basic choice paradigms for electrode placement as well as new exploratory targets, mechanisms of neuromodulation using this technique and new developments, including focused ultrasound-driven ablative procedures.

## CHOOSING TARGETS FOR DBS IN PD

In PD, the subthalamic nucleus (STN) or the internal part of the globus pallidus (GPi) are the preferred and most effective targets for alleviating the cardinal Parkinsonian signs of bradykinesia, tremor, rigidity, as well as levodopa-induced dyskinesia (LID). 1 In addition, in selected cases, DBS electrodes can be implanted in other brain locations, such as the thalamus (e.g., for tremor dominant PD), the caudal zona incerta, and the pedunculopontine nucleus (currently an experimental target for gait problems, and postural instability). 3 4 5 6 The choice of the target adds another layer of complexity and should be discussed and decided according to nuances of clinical presentation, specialist preferences, and individualized profile to each case.

### STN and GPi

DBS of both STN and GPi have been proven to be similarly effective treatment choices for patients with PD, although subtle but important outcome differences exist. 7 8 9 Overall, STN DBS tends to be preferred for control of resting tremor and rigidity, allowing for a greater reduction in dopaminergic medication dose, which may be beneficial but also a potential source of worsening of axial motor signs and certain nonmotor symptoms. As such, STN DBS-related complications include apathy, depression, impulsivity, worsened verbal fluency, and executive dysfunction in a subset of patients. 10 11 Another variable to be considered is the fact that the STN, being a smaller target, tends to require a lower amount of electrical stimulation, potentially requiring less frequent battery replacements.

GPi DBS tends to be preferred to approach cases with disabling LID, especially in patients using relatively low doses of levodopa and a low threshold for these complications. 12 Additionally, it may be beneficial for cases with “off” dystonia, and when mood and cognition are concerning. 13 A recent meta-analysis of the clinical trials concluded that STN and GPi have very similar benefits, with STN favoring medication reduction, but GPi favoring better behavioral outcomes. 14 These findings are supported by randomized clinical trials showing that GPi and STN are equally effective targets for the treatment of PD motor symptoms and LID. 15 Other studies, however, show subtle differences, as in the case of a Dutch (NSTAPS) trial 13 comparing DBS of these targets in advanced PD patients, where, patients receiving STN DBS had a more substantial improvement in motor symptoms in the long term, than those treated with DBS of the GPi, despite no between groups statistically significant differences in quality of life or the incidence of adverse events. 15 In contrast, a different prospective randomized study of unilateral stimulation of the STN and GPi found similar effects on mood and cognition, while GPi DBS led to a more pronounced improvement in the quality of life and less negative influence on verbal fluency. 16 Finally, a trial comparing 24-month outcomes for 299 patients who underwent bilateral DBS of both targets found similar improvements in motor function, maintained at the end of the follow-up period for both targets. As expected, dopaminergic medication was decreased more for the STN DBS group, while visuomotor processing speed declined less after GPi DBS. 17

### Ventral intermediate nucleus of the thalamus (VIM)

Reflecting what was described by the pioneer study of Benabid et al. 18 in essential tremor, high-frequency stimulation of the VIM improves tremor in PD. However, as it typically does not interfere significantly with other PD cardinal signs or dyskinesia, VIM DBS has been reserved for cases of tremor-dominant PD. 11 In a long-term study following stimulation of the VIM for severe tremor, including patients with PD, essential tremor, and post-traumatic tremor, reported an initial adequate response regarding tremor suppression overall in the whole group of patients. 19 The study compared these findings with their own results in patients with PD treated with STN DBS, concluding that the latter was an adequate target due to the added improvement in other motor symptoms, such as rigidity and bradykinesia. A different study comparing thalamotomy versus thalamic DBS in 45 PD patients, revealed the same efficacy for tremor suppression from each of the two procedures at five years. 16 Similarly, the multicenter European study of VIM DBS in PD and essential tremor patients studied 73 patients with Parkinsonian tremors who underwent VIM stimulation, demonstrating that tremors in the upper and lower extremities were significantly reduced without any interference with other Parkinsonian features. 19

## EXPLORATORY TARGETS

### Pedunculopontine nucleus (PPN)

The PPN has initially gained attention as a potential target for the improvement of axial signs of PD, particularly gait impairment and freezing of gait (FOG), 3 5 which are typically late issues not adequately addressed by STN or GPi stimulation. 8 The PPN is a brainstem area involved with locomotion, sensory, and behavioral processing, connecting the basal ganglia and the spinal cord. 20 Initial experimental case series of PPN DBS in PD showed subtle changes in gait parameters, added by subjective reduction in falls in spite of no other impact on PD symptoms. 8 Combining stimulation of the PPN and STN seems to provide additional motor benefit. 3 A review commissioned by the Movement Disorders Society to evaluate the utility of stimulating the PPN across centers found evidence that this procedure improves FOG, however, the degree of improvement varied within and between centers. The evidence did not support any broader benefit on other symptoms of PD, such as limb akinesia, rigidity, or tremor, 20 and did not impact allowing for a reduction in dopaminergic therapies dose. As such, there is no consensus on proper patient selection, ideal area for stimulation within the target, how to target it accurately, need for co-stimulation of other nuclei, and long-term outcomes.

### Caudal zona incerta (cZi) / Posterior subthalamic area (PSA)

A limited number of studies found promising results from DBS of the posterior subthalamic area (PSA), including the caudal zona incerta (cZi) and prelemniscal radiation. 21 Anatomically, the zona incerta region lies dorsal and posterior to the STN and is located at the junction of basal ganglia thalamocortical and cerebellar thalamocortical circuits making it an interesting target, strongly linked to tremor pathophysiology. This rationale seems to be effectively translated into clinical evidence. For example, DBS of the cZi was performed in 14 tremor-dominant PD patients with assessments at baseline on and off medication, followed for at least one year after surgery. 21 At the 18-month follow-up, the UPDRS III score improved by 47.7%, while individual scores for contralateral tremor, rigidity, and bradykinesia were improved by 82.2%, 34.3%, and 26.7%, respectively. Another randomized trial comparing cZi stimulation with the best medical treatment found a striking benefit for tremor control despite no significant reduction in medication dosage. 22 A different study compared the UPDRS scores for patients undergoing cZi DBS with a cohort of STN DBS patients. 23 The results showed superiority of cZi stimulation over STN stimulation for improving contralateral parkinsonism signs overall. Total UPDRS score improved by 76% for cZi stimulation compared with 55% for STN, while tremor scores improved by 91% for cZi compared with 61% for STN DBS. On the other hand, differences in scores for bradykinesia, dyskinesia, and reduction in dopaminergic medication dose did not reach statistical significance. As such, cZi DBS seems to be a safe and powerful target for patients with severe Parkinsonian tremors. 21 22 23

### Nucleus basalis of Meynert (NBM DBS)

DBS of the nucleus basalis of Meynert (NBM) has been proposed as a treatment option for PD dementia (PDD) and other cognitive dysfunctions. The NBM is a discrete anatomical structure in the basal forebrain, located inferior to the posterior GPi, and provides the major source of cholinergic innervation to the cortical mantle. Lately, evidence has supported the exploration of DBS to this structure as a potential therapy for PDD. A randomized, double-blind, crossover clinical trial evaluated the results of 6 patients with PDD. 24 No serious adverse events were reported in this trial and an improvement in scores on the Neuropsychiatric Inventory was observed compared with sham stimulation and the preoperative baseline. However, no improvements were observed in the primary cognitive outcomes. Another study suggests that NBM DBS is safe and feasible, and stimulation was associated with reduced right frontal and parietal glucose metabolism ( *p* < 0.01) and increased low- and high-frequency power and functional connectivity, concluding that NBM stimulation altered neuroimaging biomarkers but without lasting cognitive improvement. 25 Further studies with larger sample sizes and control groups are necessary to understand the optimal stimulation of the NBM and to assess if DBS stabilizes or reverses cognitive decline in PD compared with the best medical therapy.

Other experimental brain targets for stimulation therapy in PD, such as CM/pf 26 of the thalamus and spinal cord 27 currently have limited evidence for efficacy.

## MECHANISMS OF DBS

Over the last few decades, findings from DBS recordings of local field potentials (LPF) and multi-unit activity have led to new insights into basal ganglia circuitry and the pathophysiology of PD. The current concept emphasizes the neuronal firing pattern and synchronized oscillatory activity. In either STN or GPi, a synchronized oscillatory activity within β frequency (13 - 30Hz) has been proposed as a neurophysiological abnormality underlying PD symptoms. 28 29 Furthermore, it has been demonstrated that this β activity in the STN is a marker of a hypodopaminergic state, 30 correlating with responsiveness to levodopa. 31 Additionally, studies suggest that this firing pattern occurs in the off state and is suppressed by administration of levodopa, as well as by high-frequency stimulation in the STN. 32 These studies of LFP recordings from the STN in PD patients after administration of levodopa or apomorphine have shown that switching from off to on state correlates with a change in the firing pattern, with a reduction of β activity, which is replaced by γ activity (60 - 80Hz). Finally, in patients who develop levodopa-induced dyskinesia, the hyperkinetic movements are associated with θ activity (4 - 11Hz) in bilateral or contralateral STN implicating this firing rate band as a potential marker of dyskinesia in PD. 33

Other than these physiological findings, distinct DBS mechanisms accounting for the positive effect of DBS have been proposed. One of such mechanisms is the functional inactivation of neuronal populations near the electrodes. This has been largely attributed to the so-called depolarization block, a state in which cells undergo depolarization with an almost complete abolishment of spontaneous action potentials. Another commonly proposed mechanism underlying the effects of DBS at high frequency is the excitation of fiber pathways in the vicinity of the electrodes (afferent and efferent projections from targeted regions and fibers *en passant* ). This is an interesting possibility, as the anterograde and retrograde propagation of action potentials along such structures may influence the physiology of brain regions projecting to or receiving projections from the original point of stimulation. 34 The behavioral consequences of exciting axonal pathways may not always be positive, as a stimulation-induced tonic-firing pattern may supplant physiologic rhythms. In certain pathological states, however, the implementation of artificial brain rhythms after stimulation may be beneficial, disrupting abnormal pathologic oscillatory patterns and improving clinical outcomes, as in the example of β oscillations in PD. 35

Additionally, metabolic and neurochemical changes in structures at a distance from the target have also been suggested to contribute to DBS's mechanisms in different contexts related to this treatment modality. 34 35

## POSTOPERATIVE MANAGEMENT

Despite long-term experience with DBS in PD, there is a considerable lack of formal universal guidelines for the management of stimulation and medication in the postoperative periods. Most expert centers, however, follow standardized algorithms that often vary between institutions.

### Timing and schedule for DBS programming

The optimal moment to start the stimulation process may vary between centers with some starting the day after surgery while the patient is still admitted, while others wait until the insertional/lesional effect, which can last up to two months, subsides. The latter is probably the most common approach as this lesional effect induced by changes in tissue impedance around the electrode may interfere with the parameters used to determine the window between therapeutic and adverse effects. This is a cornerstone to start effective and successful DBS treatment, serving also as guidance on chronic parameters and future adjustments. As such, a reasonable time interval between surgery and the beginning of therapy ranges between 4 to 8 weeks. 36

Accordingly, chronic stimulation should be started at the contacts with the lowest thresholds for clinical benefit and the highest thresholds for adverse effects. Pulse width and rate (frequency) values should be kept constant and, despite the fact that there are no evidence-based guidelines to define these specific parameters, most centers use a frequency of 130Hz and pulse width of 60 μs, which are effective for the majority of cases. In PD, stimulation should start with low amplitudes, which are gradually increased in the subsequent weeks. The typical initial montage should be in a monopolar pattern with the case as anode and one active contact as a cathode, which provides a spherical electrical field around the selected electrode. A bipolar configuration (one contact as a cathode and another as anode) should be used to narrow the electrical field, avoiding the spread of the current and consequent adverse effects. 37 More advanced settings are possible, including double-monopolar, interleaving, and guarded cathode, which are rarely used in first programming sessions. Other strategies can be used for initial programming, for instance with imaging guidance and volume of tissue activation models and based on sensing of β activity from devices with LFP detection technology. These alternative forms have not yet been widely adopted. 37

It is highly recommended that after initial settings are activated, patients take their first regular dose of dopaminergic drugs and wait in the clinic until peak dose effect is reached. This will allow the observation of acute but non-immediate adverse effects such as severe dyskinesia induced by the combination of stimulation and medication.

The most common stimulation-induced complications observed during programming sessions include worsening of axial symptoms, dyskinesias, speech dysfunction, oculomotor/periorbital muscle activation, and contractures due to internal capsule stimulation. 38 These symptoms can be minimized or avoided by standard adjustment in stimulation parameters depending on specific situations. As mentioned above, stimulation-induced dyskinesias are common after STN DBS and can be minimized by different stimulation strategies or medication adjustment, while, on a positive note, they can be seen as indirect signs of accurate electrode placement. 39

### Medication management

The initial medication adjustment is performed in parallel with the initial programming sessions by a neurologist familiar with the management of PD and DBS. Again, there are no rigid formal guidelines for the management of medication after DBS. In patients with STN DBS, a reduction in dopaminergic therapy of 40 to 60% is usually achieved within weeks or a few months after surgery. 40 The reduction in PD therapy dosage begins when the amplitude of stimulation is increased to a level that provides symptomatic relief of motor symptoms and fluctuations, which often happens following the second programming visit. The sequence and form of medication reduction are usually individualized according to the patient's symptoms, profile, and regimen. It is reasonable to focus on one drug class at a time to minimize adverse reactions and withdrawal symptoms. 41 For example, anticholinergics and MAO inhibitors can be the first to be titrated down, followed by COMT inhibitors, amantadine, and then, reduction in levodopa and dopamine agonist. 42 Again, this sequence is a generic suggestion that needs to be carefully revised individually and includes many exceptions. For instance, in patients with a history of impulse control disorders, dopamine agonists are usually discontinued with great caution earlier after surgery.

During this phase, patients should be closely monitored, not only due to the risk of early medication withdrawal but also given the occurrence of behavioral changes (depression, suicide risk, apathy, fatigue, etc.) associated with pronounced and abrupt medication reduction, downregulating mesolimbic dopaminergic denervation. 40 41 42 43 A low dose of dopamine agonist has shown to be effective in managing withdrawal hypodopaminergic behaviors in this setting. 42 Conversely, some patients tend to develop hyperdopaminergic behaviors such as euphoria and hypomanic states, which seem to be more associated with the surgical procedure itself and the location of the stimulating contacts within the limbic segments of the STN. 44

## ADVANCES IN DBS THERAPY

Recent advances in technology and technique have continuously been implemented in new generations of DBS systems, increasing therapeutic yield and improving management of adverse effects, reducing patient burden, and clinician programming times. These advances include current delivery devices, directional leads, new exploratory targets, programming approaches based on the volume of tissue activation models, insertion techniques, and identification of better biomarkers for patient selection and prediction of outcomes.

### Directional stimulation

Conventional DBS systems utilize ring-shaped electrodes, which produce omnidirectional spherical electric fields with little additional control of the shape of the volume of tissue activated. Directional leads differ in that they allow for horizontal current steering via changes in electrode design, allowing the electric field to deviate away from structures that induce undesirable side effects. Of importance, these systems place directional leads at the two middle contact levels, while the most ventral and the most dorsal ones retain the ring-mode design distributing the current radially. 45 46

These advances in technology come at a cost. For instance, an increase in patient's expectations gives the false impression that the device can allow for an unrestricted fix of stimulation and disease-related problems. Also, despite the fact that the current steering capability allows for some degree of rescue of the therapeutic effect of misplaced electrodes, precise targeting of brain nuclei should continue to be the goal. Finally, it should be kept in mind that programming is becoming increasingly more sophisticated and challenging in terms of demand for time, complexity, and need for advanced expertise. The real advantage of this technological advance needs to be firmly confirmed in clinical practice.

### Adaptive or closed-loop stimulation

The existent model of DBS involves continuous stimulation input, which does not consider real-time pathophysiological phenomena recorded using LFP of the brain areas being stimulated, such as β oscillatory activity, a marker of the therapeutic effect of levodopa. In the adaptive DBS paradigm, DBS is activated based on electrophysiological feedback parameters indicating its need. This approach was initially tested on a primate animal model of PD using closed-loop stimulation of the GPi based on ongoing activity in M1, an approach that proved to be more efficient overall in alleviating motor symptoms than traditional open-loop stimulation. 47 Besides, a greater reduction of pathological oscillatory activity in the pallidum and primary motor cortex with closed-loop stimulation versus open-loop stimulation was documented. 47 Another study found that a small sample of PD patients who underwent closed-loop stimulation had significantly greater improvement of the PD cardinal symptoms with fewer adverse effects, as compared with when they underwent open-loop stimulation. 48

In summary, the limited but consistent body of literature regarding this technique promises considerable advantages (reduced adverse effects, optimal clinical benefit stimulation delivery to match the patient's fluctuations, and extended battery life) and also allows us to improve our understanding of functional (and malfunctioning) neurocircuitry. 49

### Interleaving stimulation

The technique of interleaved stimulation entails running two programs with different settings on the same lead in a temporally alternating sequence, dictated by the programmed frequency. This allows for additional flexibility in creating and reshaping the electrical field along the longitudinal axis of a multi-contact ring electrode, keeping in mind that it does not allow horizontal current steering in any shape or form. 50 The utility of this programming variation can be theoretically useful in two scenarios:

^•^ to limit stimulation-induced adverse effects (reshaping of the electric field) and

^•^ stimulation of different brain regions with individualized settings of amplitude and pulse width to alleviate symptoms and signs that are driven by specific and separate areas around the core of the stimulated nuclei (i.e., stimulation of the ventral and dorsal STN to control dyskinesias and parkinsonism). 51 52 53

### MRI-guided and CT-verified DBS implantation in asleep patients

Awake intraoperative microelectrode recording combined with macro stimulation is the most common technique used by most centers to compensate for imaging limitations and subtle stereotactic incongruences needed for optimized electrode placement. On the other hand, awake mapping can be poorly tolerated by a minority of DBS surgery candidates due to claustrophobia, anxiety, high-amplitude tremor, or painful dystonic posturing. 54 In these instances, considering DBS with general anesthesia has emerged as an option. 55 This technique often is coupled with real-time intraoperative MRI or CT scanning, while commercially available options for intraoperative imaging are gaining visibility including operating rooms with mobile magnets, 56 and implantation using a CT-guided approach. 54 55 56 57 However, the safety and clinical outcome of this technique remains a matter of debate. 57 58 In a single-center study, the authors found no difference in complication rates between asleep and awake DBS, but the study did not evaluate the motor outcomes between the two cohorts. 59 60 Similarly, a study using frame-based DBS implantation under general anesthesia with intraoperative MRI verification of lead location demonstrated that this technique is safe, precise, accurate, and effective compared with standard implantation performed using awake intraoperative physiology. 61 However, another study suggested that there might be a lower overall intraoperative complication rate with asleep DBS but at the cost of a higher rate of postoperative stimulation-related complications compared with awake DBS.

## MR-GUIDED FOCUSED ULTRASOUND

The potential effect of focused ultrasound (FUS) in heating live tissues was first recognized when high-intensity ultrasound waves were used in submarines during World War II, as it was noticed that it could heat up and kill marine life. 62 In the early 1950s, FUS was experimentally tested on body tissues as an alternative to ablative procedures, including lung and brain. 63 At the time, the technique was more “invasive,” requiring a craniotomy to allow the ultrasound energy to reach the target, ultimately leading to the understanding that it was not overall more advantageous compared with traditional radiofrequency ablative surgery. 64 65 Over the decades since then, steady advances in technology allowed accurate targeting of deeper structures in the human body, eventually to leading the current use of the magnetic resonance-guided focused ultrasound (MRgFUS) technique as a form of non-invasive stereotactic ablative procedure on a wide range of indications. 66

The principle of FUS relies on its ability to induce cytotoxic levels of increased tissue temperature with a relatively low risk of vascular vulnerability. As a consequence, this technique can be more precise than others, such as gamma knife radiosurgery, culminating with the submillimeter precision capability of modern devices. 65 As such, based on solid and sound scientific evidence, MRgFUS has been approved by various health agencies worldwide for the treatment of essential tremor (ET) and, more recently, PD.

The first series of MRgFUS thalamotomy in tremor-dominant PD was published by Schlesinger et al. in 2015. 63 This pilot study of seven cases followed for up to 12 months (mean 7.3 months) showed mean PDQ-39 and total UPDRS scores improvement by almost 50% for both scales comparing preoperative and 1 week postoperatively. Three of these cases presented subtle reemergence of tremor at 1 week, 1 month, and 6 months after the procedure, respectively. Otherwise, there were no permanent adverse effects. The same group published another study with 12 tremor-dominant PD cases in 2018, now with a longer follow-up (mean 11.5, ranging from 6–24 months). 64 Again, mean motor UPDRS and PDQ-39 scores improvements were significant, reaching 46.2% and 46.6%, respectively, after 6 months postoperatively. A third of these cases experienced tremor recurrence within 6 months after the procedure, which continued to be significantly less disabling compared with preoperative baseline. Fasano et al. 65 published a single-blinded prospective study of three tremor-dominant PD cases with follow-up of up to six months. The reduction in tremor scores was in the range of 50%, inducing an improvement of 32.3% in the activities of daily living section of the UPDRS from baseline to the latest follow-up. Both mild, transient, and more significant and long-lasting complications were observed in single cases, in addition to tremor recurrence in one of these cases. In the same year, a double-blinded, sham-controlled randomized trial of MRgFUS thalamotomy in tremor-dominant PD was published, including 27 patients reporting a 62% improvement from baseline to 3 months postoperatively in tremor scores, compared with 22% in the sham group. Persistent adverse events of thalamic lesion included paresthesia in 19% and ataxia in 4%, 67 in STN adverse events can occur as dyskinesias, weakness, speech, and gait disturbance which in the majority of the cases are transient and last for a few weeks. 68

The first report of unilateral MRgFUS pallidotomy for PD was published in 2015, of a 55 years old patient with severe motor fluctuations and levodopa-induced dyskinesias. The outcomes at up to 6 months were very significant, including a reduction of 61% in the UPDRS motor scores and of 76% in dyskinesia (UPDRS part IV) scores after 6 months. 69 Later, a series of 10 cases of PD treated with unilateral MRgFUS pallidotomy was published in 2018 with peak dose dyskinesias severity as the main treatment outcome. Patients were followed for 12 months with significant improvements of 32.2% in the off-medication condition motor scores and 52.7% in dyskinesia scores, which correlated with improvement in quality of life. None presented persistent adverse effects. 70 Although the worldwide experience with this technique and target in PD is growing, the overall impression is that it compares to radiofrequency pallidotomy in terms of effectiveness but may be more advantageous from the standpoint of safety.

Unilateral MRgFUS subthalamotomy has only been studied in one open trial with a series of 10 PD patients showing an improvement of 53% in motor UPDRS scores on the contralateral body side in the off-medication condition 6 months after the treatment. One patient developed transient contralateral hyperkinetic movements after the procedure. 69 This is a promising target for MRgFUS in PD, however, most of the exiguous literature on this specific topic and target comes from a single center and requires further exploration. A comparison between MRgFUS and DBS can be found in Table 1 .

**Table 1:** Comparing MRgFUS and DBS

MRgFUS	DBS
Clinically used in PD since 2018 with limited experience and availability	Clinically used in PD since 1994 with broad worldwide experience
Stereotactic frame needed	Stereotactic frame needed
No incision needed	Incision needed
One step procedure	One or two steps procedure
No implantable device needed	Implantable device needed
Currently unilateral	Bilateral procedure, if indicated
Lower incidence of acute complications	Higher risk (+/− 5%) of acute complications
Effect depends on lesional effect (ablation)	Effect depends on electrical stimulation /transient microlesional effect
Irreversible	Reversible
Immediate therapeutic effect	Therapeutic effect requires stimulation onset and optimization

In conclusion, DBS has become an established therapy for PD over the past decades, with good quality evidence for STN and GPi as the primary targets to address PD cardinal signs, motor fluctuations, and LID. Therapy with DBS is an ongoing process of refinement and sophistication in terms of technical development and in several clinical aspects such as selection of patients, choice of targets, and exploration of stimulation paradigms. One of the main dogmatic changes in the field was its consideration for patients with shorter disease duration, after motor complications develop, instead of later on, when the disease is too advanced, and results are suboptimal. As a dynamic field, further developments are expected in the near future, hopefully broadening these options for physicians and patients. Despite all the progress, access to DBS therapy is still limited in developing countries and remote areas, either due lack of awareness and scarce financial, logistic, and manpower resources. This review aimed to help fill in the knowledge gap, covering the fundamental aspects of DBS for the management of PD.

## References

[1] Strauss I Kalia S K Lozano A M Where are we with surgical therapies for Parkinson's disease? Parkinsonism Relat Disord 2014 20 01 S187 S191 10.1016/S1353-8020(13)70044-0 24262178

[2] Vedam-Mai V Deisseroth K Giordano JProceedings of the Eighth Annual Deep Brain Stimulation Think Tank: Advances in Optogenetics, Ethical Issues Affecting DBS Research, Neuromodulatory Approaches for Depression, Adaptive Neurostimulation, and Emerging DBS Technologies. Front Hum Neurosci. 2021;15:644593. 10.3389/fnhum.2021.644593 PMC8092047 33953663

[3] Khan S Mooney L Plaha P Outcomes from stimulation of the caudal zona incerta and pedunculopontine nucleus in patients with Parkinson's disease Br J Neurosurg 2011 25 02 273 280 10.3109/02688697.2010.544790 21344974

[4] Stefani A Lozano A M Peppe A Bilateral deep brain stimulation of the pedunculopontine and subthalamic nuclei in severe Parkinson's disease Brain 2007 130(Pt 6):1596 1607 10.1093/brain/awl346 17251240

[5] Khan S Javed S Mooney L Clinical outcomes from bilateral versus unilateral stimulation of the pedunculopontine nucleus with and without concomitant caudal zona incerta region stimulation in Parkinson's disease Br J Neurosurg 2012 26 05 722 725 10.3109/02688697.2012.659297 22404735

[6] Weiss D Walach M Meisner C Nigral stimulation for resistant axial motor impairment in Parkinson's disease? A randomized controlled trial Brain 2013 136(Pt 7):2098 2108 10.1093/brain/awt122 23757762 PMC3692032

[7] Bronstein J M Tagliati M Alterman R L Deep brain stimulation for Parkinson disease: an expert consensus and review of key issuesIn: Vol 68.2011 165 10.1001/archneurol.2010.260 PMC4523130 20937936

[8] Follett K A Torres-Russotto D Deep brain stimulation of globus pallidus interna, subthalamic nucleus, and pedunculopontine nucleus for Parkinson's disease: which target? Parkinsonism Relat Disord 2012 18 01 S165 S167 10.1016/S1353-8020(11)70051-7 22166422

[9] Lukins T R Tisch S Jonker B The latest evidence on target selection in deep brain stimulation for Parkinson's disease J Clin Neurosci 2014 21 01 22 27 10.1016/j.jocn.2013.05.011 24210797

[10] Multicentre Advanced Parkinson's Disease Deep Brain Stimulation Group Hariz M I Rehncrona S Quinn N P Speelman J D Wensing C Multicenter study on deep brain stimulation in Parkinson's disease: an independent assessment of reported adverse events at 4 years Mov Disord 2008 23 03 416 421 10.1002/mds.21888 18067188

[11] Mansouri A Taslimi S Badhiwala J H Deep brain stimulation for Parkinson's disease: meta-analysis of results of randomized trials at varying lengths of follow-up J Neurosurg 2018 128 04 1199 1213 10.3171/2016.11.JNS16715 28665252

[12] Munhoz R P Cerasa A Okun M S Surgical treatment of dyskinesia in Parkinson's disease Front Neurol 2014 5 65 10.3389/fneur.2014.00065 24808889 PMC4010755

[13] Odekerken V JJ van Laar T Staal M J Subthalamic nucleus versus globus pallidus bilateral deep brain stimulation for advanced Parkinson's disease (NSTAPS study): a randomised controlled trial Lancet Neurol 2013 12 01 37 44 10.1016/S1474-4422(12)70264-8 23168021

[14] NSTAPS study group Odekerken V JJ Boel J A Schmand B A GPi vs STN deep brain stimulation for Parkinson disease: Three-year follow-up Neurology 2016 86 08 755 761 10.1212/WNL.0000000000002401 26819458

[15] Okun M S Fernandez H H Wu S S Cognition and mood in Parkinson's disease in subthalamic nucleus versus globus pallidus interna deep brain stimulation: the COMPARE trial Ann Neurol 2009 65 05 586 595 10.1002/ana.21596 19288469 PMC2692580

[16] O'Sullivan D Pell M Long-term follow-up of DBS of thalamus for tremor and STN for Parkinson's disease Brain Res Bull 2009 78(2-3):119 121 10.1016/j.brainresbull.2008.09.001 18834932

[17] CSP 468 Study Group Follett K A Weaver F M Stern M Pallidal versus subthalamic deep-brain stimulation for Parkinson's disease N Engl J Med 2010 362 22 2077 2091 10.1056/NEJMoa0907083 20519680

[18] Benabid A L Pollak P Gervason C Long-term suppression of tremor by chronic stimulation of the ventral intermediate thalamic nucleus Lancet 1991 337(8738):403 406 10.1016/0140-6736(91)91175-t 1671433

[19] Limousin P Speelman J D Gielen F Janssens M Multicentre European study of thalamic stimulation in parkinsonian and essential tremor J Neurol Neurosurg Psychiatry 1999 66 03 289 296 10084526 10.1136/jnnp.66.3.289 PMC1736277

[20] Nowacki A Galati S Ai-Schlaeppi J Bassetti C Kaelin A Pollo C Pedunculopontine nucleus: An integrative view with implications on Deep Brain Stimulation Neurobiol Dis 2019 128 75 85 10.1016/j.nbd.2018.08.015 30189263

[21] Blomstedt P Fytagoridis A Åström M Linder J Forsgren L Hariz M I Unilateral caudal zona incerta deep brain stimulation for Parkinsonian tremor Parkinsonism Relat Disord 2012 18 10 1062 1066 10.1016/j.parkreldis.2012.05.024 22709794

[22] Blomstedt P Stenmark Persson R Hariz G-M Deep brain stimulation in the caudal zona incerta versus best medical treatment in patients with Parkinson's disease: a randomised blinded evaluation J Neurol Neurosurg Psychiatry 2018 89 07 710 716 10.1136/jnnp-2017-317219 29386253 PMC6031280

[23] Plaha P Ben-Shlomo Y Patel N K Gill S S Stimulation of the caudal zona incerta is superior to stimulation of the subthalamic nucleus in improving contralateral parkinsonism Brain 2006 129(Pt 7):1732 1747 10.1093/brain/awl127 16720681

[24] Gratwicke J Zrinzo L Kahan J Bilateral Deep Brain Stimulation of the Nucleus Basalis of Meynert for Parkinson Disease Dementia: A Randomized Clinical Trial JAMA Neurol 2018 75 02 169 178 10.1001/jamaneurol.2017.3762 29255885 PMC5838617

[25] Sasikumar S Cohn M Harmsen I E Single-Trajectory Multiple-Target Deep Brain Stimulation for Parkinsonian Mobility and Cognition Mov Disord 2022 37 03 635 640 10.1002/mds.28870 34806782

[26] Caparros-Lefebvre D Blond S Feltin M P Pollak P Benabid A L Improvement of levodopa induced dyskinesias by thalamic deep brain stimulation is related to slight variation in electrode placement: possible involvement of the centre median and parafascicularis complex J Neurol Neurosurg Psychiatry 1999 67 03 308 314 10.1136/jnnp.67.3.308 10449551 PMC1736532

[27] Fonoff E T de Lima-Pardini A C Coelho D B Spinal Cord Stimulation for Freezing of Gait: From Bench to Bedside Front Neurol 2019 10 905 10.3389/fneur.2019.00905 31507514 PMC6718563

[28] Brown P Oliviero A Mazzone P Insola A Dopamine Dependency of Oscillations between Subthalamic Nucleus and Pallidum in Parkinson's Disease The Journal of …. January 2001 1 6 http://www.jneurosci.org/content/21/3/1033.short 10.1523/JNEUROSCI.21-03-01033.2001 PMC6762327 11157088

[29] Özkurt T E Butz M Homburger M High frequency oscillations in the subthalamic nucleus: a neurophysiological marker of the motor state in Parkinson's disease Exp Neurol 2011 229 02 324 331 10.1016/j.expneurol.2011.02.015 21376039

[30] Levy R Ashby P Hutchison W D Lang A E Lozano A M Dostrovsky J O Dependence of subthalamic nucleus oscillations on movement and dopamine in Parkinson's disease Brain 2002 125(Pt 6):1196 1209 12023310 10.1093/brain/awf128

[31] Weinberger M Mahant N Hutchison W D Beta oscillatory activity in the subthalamic nucleus and its relation to dopaminergic response in Parkinson's disease J Neurophysiol 2006 96 06 3248 3256 10.1152/jn.00697.2006 17005611

[32] Kühn A A Kempf F Brücke C High-frequency stimulation of the subthalamic nucleus suppresses oscillatory beta activity in patients with Parkinson's disease in parallel with improvement in motor performance J Neurosci 2008 28 24 6165 6173 10.1523/JNEUROSCI.0282-08.2008 18550758 PMC6670522

[33] Alonso-Frech F Zamarbide I Alegre M Slow oscillatory activity and levodopa-induced dyskinesias in Parkinson's disease Brain 2006 129(Pt 7):1748 1757 10.1093/brain/awl103 16684788

[34] Brown P Oscillatory nature of human basal ganglia activity: relationship to the pathophysiology of Parkinson's disease Mov Disord 2003 18 04 357 363 10.1002/mds.10358 12671940

[35] Ashkan K Rogers P Bergman H Ughratdar I Insights into the mechanisms of deep brain stimulation Nat Rev Neurol 2017 13 09 548 554 10.1038/nrneurol.2017.105 28752857

[36] Volkmann J Moro E Pahwa R Basic algorithms for the programming of deep brain stimulation in Parkinson's disease Mov Disord 2006 21 14 S284 S289 10.1002/mds.20961 16810675

[37] Munhoz R P Albuainain G Deep brain stimulation: new programming algorithms and teleprogramming Expert Rev Neurother 2023 23 05 467 478Epub ahead of print 10.1080/14737175.2023.2208749 37115193

[38] Picillo M Lozano A M Kou N Puppi Munhoz R Fasano A Programming Deep Brain Stimulation for Parkinson's Disease: The Toronto Western Hospital Algorithms Brain Stimul 2016 9 03 425 437 10.1016/j.brs.2016.02.004 26968806

[39] Sriram A Foote K D Oyama G Kwak J Zeilman P R Okun M S Brittle Dyskinesia Following STN but not GPi Deep Brain Stimulation Tremor Other Hyperkinet Mov (N Y) 2014 4 242 10.7916/D8KS6PPR 24932426 PMC4050173

[40] Castrioto A Lhommée E Moro E Krack P Mood and behavioural effects of subthalamic stimulation in Parkinson's disease Lancet Neurol 2014 13 03 287 305 10.1016/S1474-4422(13)70294-1 24556007

[41] Thobois S Ardouin C Lhommée E Non-motor dopamine withdrawal syndrome after surgery for Parkinson's disease: predictors and underlying mesolimbic denervation Brain 2010 133(Pt 4):1111 1127 10.1093/brain/awq032 20237128

[42] Thobois S Lhommée E Klinger H Parkinsonian apathy responds to dopaminergic stimulation of D2/D3 receptors with piribedil Brain 2013 136(Pt 5):1568 1577 10.1093/brain/awt067 23543483

[43] Voon V Krack P Lang A E A multicentre study on suicide outcomes following subthalamic stimulation for Parkinson's disease Brain 2008 131(Pt 10):2720 2728 10.1093/brain/awn214 18941146 PMC2724899

[44] Welter M-L Schüpbach M Czernecki V Optimal target localization for subthalamic stimulation in patients with Parkinson disease Neurology 2014 82 15 1352 1361 10.1212/WNL.0000000000000315 24647024 PMC4001189

[45] Contarino M F Bour L J Verhagen R Directional steering: A novel approach to deep brain stimulation Neurology 2014 83 13 1163 1169 10.1212/WNL.0000000000000823 25150285

[46] Pollo C Kaelin-Lang A Oertel M F Directional deep brain stimulation: an intraoperative double-blind pilot study Brain 2014 137(Pt 7):2015 2026 10.1093/brain/awu102 24844728

[47] Rosin B Slovik M Mitelman R Closed-loop deep brain stimulation is superior in ameliorating parkinsonism Neuron 2011 72 02 370 384 10.1016/j.neuron.2011.08.023 22017994

[48] Little S Tripoliti E Beudel M Adaptive deep brain stimulation for Parkinson's disease demonstrates reduced speech side effects compared to conventional stimulation in the acute setting J Neurol Neurosurg Psychiatry 2016 87 12 1388 1389 10.1136/jnnp-2016-313518 27530809 PMC5136720

[49] Little S Brown P Debugging Adaptive Deep Brain Stimulation for Parkinson's Disease Mov Disord 2020 35 04 555 561 10.1002/mds.27996 32039501 PMC7166127

[50] Miocinovic S Khemani P Whiddon R Outcomes, management, and potential mechanisms of interleaving deep brain stimulation settings Parkinsonism Relat Disord 2014 20 12 1434 1437 10.1016/j.parkreldis.2014.10.011 25457819

[51] Zhang S Zhou P Jiang S Wang W Li P Interleaving subthalamic nucleus deep brain stimulation to avoid side effects while achieving satisfactory motor benefits in Parkinson disease: A report of 12 cases Medicine (Baltimore) 2016 95 49 e5575 10.1097/MD.0000000000005575 27930569 PMC5266041

[52] Kern D S Picillo M Thompson J A Interleaving Stimulation in Parkinson's Disease, Tremor, and Dystonia Stereotact Funct Neurosurg 2018 96 06 379 391 10.1159/000494983 30654368

[53] Aquino C C Duffley G Hedges D M Interleaved deep brain stimulation for dyskinesia management in Parkinson's disease Mov Disord 2019 34 11 1722 1727 10.1002/mds.27839 31483534 PMC10957149

[54] Matias C M Frizon L A Nagel S J Lobel D A Machado A G Deep brain stimulation outcomes in patients implanted under general anesthesia with frame-based stereotaxy and intraoperative MRI J Neurosurg 2018 129 06 1572 1578 10.3171/2017.7.JNS171166 29372880

[55] Martin A J Larson P S Ostrem J L Starr P A Interventional magnetic resonance guidance of deep brain stimulator implantation for Parkinson disease Top Magn Reson Imaging 2009 19 04 213 221 10.1097/RMR.0b013e3181963b2c 19148038

[56] Rahmathulla G Recinos P F Traul D E Surgical briefings, checklists, and the creation of an environment of safety in the neurosurgical intraoperative magnetic resonance imaging suite Neurosurg Focus 2012 33 05 E12 10.3171/2012.9.FOCUS12260 23116092

[57] Chen T Mirzadeh Z Chapple K Lambert M Dhall R Ponce F A “Asleep” deep brain stimulation for essential tremor J Neurosurg 2016 124 06 1842 1849 10.3171/2015.6.JNS15526 26613177

[58] Saleh S Swanson K I Lake W B Sillay K A Awake Neurophysiologically Guided versus Asleep MRI-Guided STN DBS for Parkinson Disease: A Comparison of Outcomes Using Levodopa Equivalents Stereotact Funct Neurosurg 2015 93 06 419 426 10.1159/000442425 26784455

[59] Nakajima T Zrinzo L Foltynie T MRI-guided subthalamic nucleus deep brain stimulation without microelectrode recording: can we dispense with surgery under local anaesthesia? Stereotact Funct Neurosurg 2011 89 05 318 325 10.1159/000330379 21921673

[60] Chen T Mirzadeh Z Chapple K Lambert M Ponce F A Complication rates, lengths of stay, and readmission rates in “awake” and “asleep” deep brain simulation J Neurosurg 2017 127 02 360 369 10.3171/2016.6.JNS152946 27662532

[61] Ho A L Ali R Connolly I D Awake versus asleep deep brain stimulation for Parkinson's disease: a critical comparison and meta-analysis J Neurol Neurosurg Psychiatry 2018 89 07 687 691 10.1136/jnnp-2016-314500 28250028

[62] Jagannathan J Sanghvi N K Crum L A Yen C P Ricky Medel, Aaron S Dumont, Sheehan JP, Steiner L, Jolesz F, Kassell NF. High intensity focused ultrasound surgery (HIFU) of the brain: A historical perspective, with modern applications Neurosurgery 2009 64 02 201 211 19190451 10.1227/01.NEU.0000336766.18197.8E PMC4068031

[63] Schlesinger I Eran A Sinai A MRI Guided Focused Ultrasound Thalamotomy for Moderate-to-Severe Tremor in Parkinson's Disease Parkinsons Dis 2015 2015 219149 26421209 10.1155/2015/219149 PMC4572440

[64] Zaaroor M Sinai A Goldsher D Eran A Nassar M Schlesinger I Magnetic resonance-guided focused ultrasound thalamotomy for tremor: a report of 30 Parkinson's disease and essential tremor cases J Neurosurg 2018 128 01 202 210 28298022 10.3171/2016.10.JNS16758

[65] Fasano A Llinas M Munhoz R P Hlasny E Kucharczyk W Lozano A M MRI-guided focused ultrasound thalamotomy in non-ET tremor syndromes Neurology 2017 89 08 771 775 28747452 10.1212/WNL.0000000000004268

[66] Bond A E Shah B B Huss D S Safety and Efficacy of Focused Ultrasound Thalamotomy for Patients With Medication-Refractory, Tremor-Dominant Parkinson Disease: A Randomized Clinical Trial JAMA Neurol 2017 74 12 1412 1418 29084313 10.1001/jamaneurol.2017.3098 PMC5822192

[67] Na Y C Chang W S Jung H H Kweon E J Chang J W Unilateral magnetic resonance-guided focused ultrasound pallidotomy for Parkinson disease Neurology 2015 85 06 549 551 26180137 10.1212/WNL.0000000000001826

[68] Martínez-Fernández R Máñez-Miró J U Rodríguez-Rojas R Randomized trial of focused ultrasound subthalamotomy for Parkinson's disease N Engl J Med 2020 383 26 2501 2513 33369354 10.1056/NEJMoa2016311

[69] Jung N Y Park C K Kim M Lee P H Sohn Y H Chang J W The efficacy and limits of magnetic resonance-guided focused ultrasound pallidotomy for Parkinson's disease: a Phase I clinical trial J Neurosurg 2018 ••• 1 9 10.3171/2018.2.JNS172514 30095337

[70] Martínez-Fernández R Rodríguez-Rojas R Del Álamo M Focused ultrasound subthalamotomy in patients with asymmetric Parkinson's disease: a pilot study Lancet Neurol 2018 17 01 54 63 29203153 10.1016/S1474-4422(17)30403-9

